# Identifying Prognostic Features by Bottom-Up Approach and Correlating to Drug Repositioning

**DOI:** 10.1371/journal.pone.0118672

**Published:** 2015-03-04

**Authors:** Wei Li, Jian Yu, Baofeng Lian, Han Sun, Jing Li, Menghuan Zhang, Ling Li, Yixue Li, Qian Liu, Lu Xie

**Affiliations:** 1 Key Laboratory of Biomedical Photonics of Ministry of Education, College of Life Science and Technology, Huazhong University of Science and Technology, Wuhan, 430074, P. R. China; 2 Shanghai Center for Bioinformation Technology, Shanghai Institutes of Biomedicine, Shanghai Academy of Science and Technology, Shanghai, 201203, P. R. China; 3 Britton Chance Center for Biomedical Photonics, Wuhan National Laboratory for Optoelectronics, Huazhong University of Science and Technology, Wuhan, 430074, P. R. China; 4 Department of Bioinformatics and Biostatistics, Shanghai Jiaotong University, Shanghai, 200240, P. R. China; 5 Key Laboratory of Systems Biology, Chinese Academy of Sciences, Shanghai, 200031, P. R. China; Institute of Molecular and Cell Biology, Biopolis, UNITED STATES

## Abstract

**Background:**

Traditionally top-down method was used to identify prognostic features in cancer research. That is to say, differentially expressed genes usually in cancer versus normal were identified to see if they possess survival prediction power. The problem is that prognostic features identified from one set of patient samples can rarely be transferred to other datasets. We apply bottom-up approach in this study: survival correlated or clinical stage correlated genes were selected first and prioritized by their network topology additionally, then a small set of features can be used as a prognostic signature.

**Methods:**

Gene expression profiles of a cohort of 221 hepatocellular carcinoma (HCC) patients were used as a training set, ‘bottom-up’ approach was applied to discover gene-expression signatures associated with survival in both tumor and adjacent non-tumor tissues, and compared with ‘top-down’ approach. The results were validated in a second cohort of 82 patients which was used as a testing set.

**Results:**

Two sets of gene signatures separately identified in tumor and adjacent non-tumor tissues by bottom-up approach were developed in the training cohort. These two signatures were associated with overall survival times of HCC patients and the robustness of each was validated in the testing set, and each predictive performance was better than gene expression signatures reported previously. Moreover, genes in these two prognosis signature gave some indications for drug-repositioning on HCC. Some approved drugs targeting these markers have the alternative indications on hepatocellular carcinoma.

**Conclusion:**

Using the bottom-up approach, we have developed two prognostic gene signatures with a limited number of genes that associated with overall survival times of patients with HCC. Furthermore, prognostic markers in these two signatures have the potential to be therapeutic targets.

## Introduction

Hepatocellular carcinoma (HCC) is the third leading cause of cancer-related death in the world, especially in Asia and Africa[1]. Surgical resection is one of the most important curative treatments for HCC, while long-term survival of HCC remains poor because of high recurrence rate. Improvements in early diagnosis and accurate staging systems can help guide patients to take optimum treatment strategies that may suppress recurrence and prolong survival[2].

Currently, beyond tumor-node-metastasis(TNM) staging system, several prognostic algorithms used to predict survival among patients with hepatocellular carcinoma have been established, Barcelona Clinic Liver Cancer (BCLC) and Cancer of the Liver Italian Program (CLIP) systems are among the most commonly used systems worldwide[3–7]. Nonetheless, all these staging systems only select optional clinical and serum biochemical indexes such as tumor size, vascular invasion, alpha fetoprotein (AFP), albumin(ALB), etc. Although these clinicopathologic staging systems have been proven useful, their predictive accuracy remains limited and they failed to provide molecular biological characteristics of HCC that might be genetic and heterogenic. With the recent advances in genome studies, gene expression profiling-based studies have improved our understanding of cancer biology and gene expression signatures have been successfully used as prognostic tools especially in breast cancer[8–10].

Recently, two main strategies have been used for prognostic gene signature identification, symbolized as “top-down” or “bottom-up”. In ‘top-down’ approach firstly genes with different expression patterns between case samples and control samples were sought, secondly gene(s) with expression level(s) significantly correlating to histological grades or biological phenotypes were selected as candidate genes, then a regression model with best predictors was built to construct the final gene signature. Many studies have applied this approach and identified quite a few gene signatures with prognostic values, such as MammaPrint[11]. Unfortunately, prognostic signatures identified by this approach in HCC present minimal overlaps and few of them have been adopted in routine clinical practice[12–14]. The ‘bottom-up’ approach was firstly based on supervised analysis of genes which are directly associated with occurrence of the event studied (metastasis, survival), Secondly some genes were selected by machine learning algorithms or significant enrichment analysis of specific pathways or biological functions, and then the prognostic value of these gene sets could be calculated[15,16]. This ‘bottom-up’ approach was applied by some groups in several cancers but few of assays used this approach to identify HCC prognostic signatures. While the candidate gene set was determined by “top-down” or “bottom-up” approach, various machine learning algorithms including regression models can be used to identify the final gene signatures. However, overfitting and the low accuracy in independent cohort limited the clinical application of these algorithms[17]. Meanwhile, analysis of networks and modular biological processes has shown the effective capacity to estimate key genes which may have impact on patient outcomes[18].

In addition to identifying prognostic signatures in tumor tissues, many research groups showed that genes in adjacent non-tumor tissues appear to be implicated in tumour progression and aggressiveness, gene expression profiles in surrounding non-tumor tissues can be helpful to identify signatures associated with outcomes[9]. This can be especially true with adjacent non-tumor tissues for HCC, where often pathological change such as cirrhosis is present because of long-term inflammation caused by hepatitis B or C virus (HBV or HCV) infection[9,19].

Over the past years, large investments have been input to discover novel markers for valuable biological insights to mechanisms of many common diseases. Nonetheless, the translation from genetic findings to clinical applications remains limited. Recently, some works explored one potential application of these genetic molecular markers: drug repositioning[20,21]. We suppose that prognostic gene signatures could not only be used for predicting patient outcome, but also can be used to gain important information for drug discovery, which can then be utilized to provide appropriate therapeutic advices to patients.

**Fig 1:**
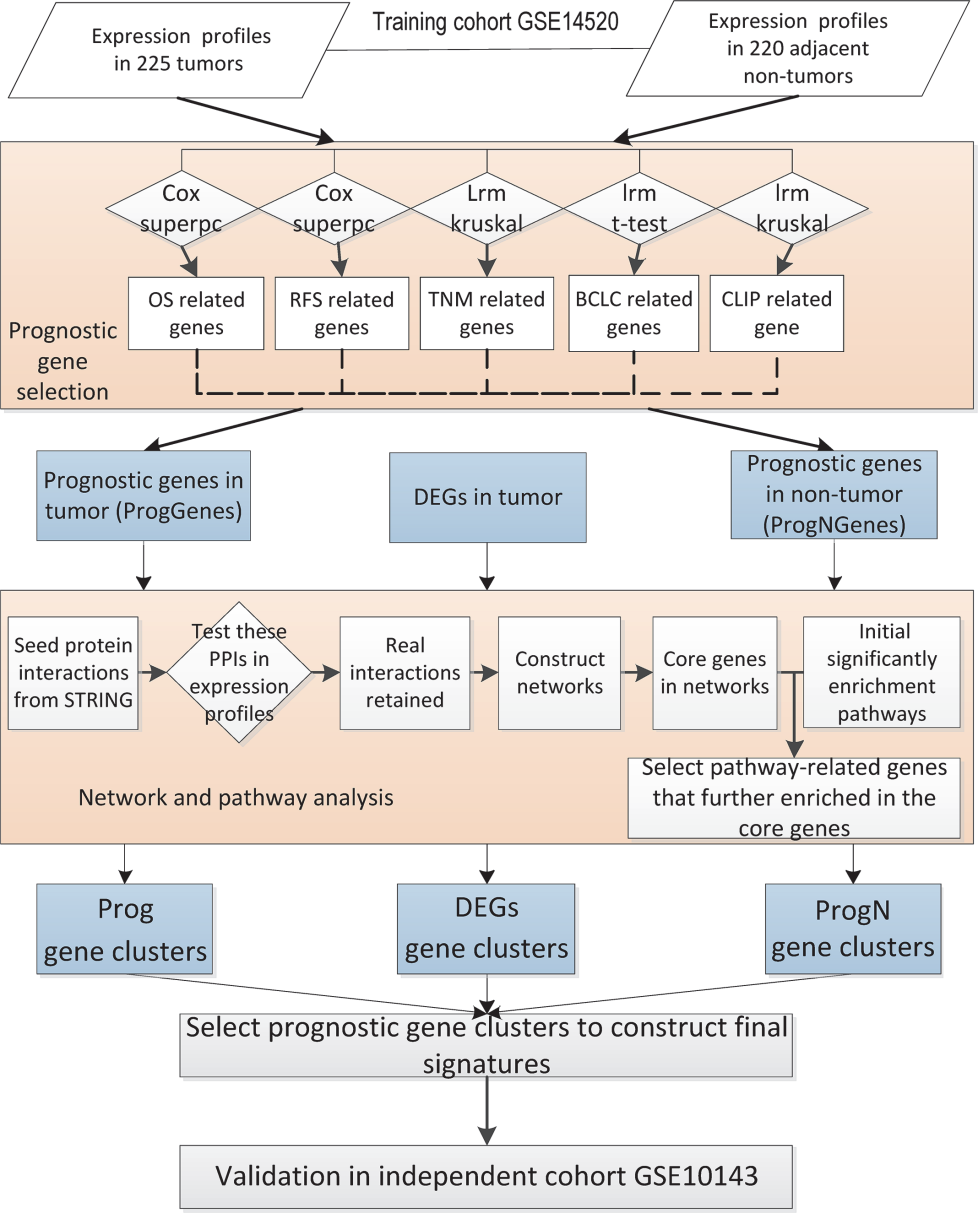
The workflow of prognostic model construction. Firstly, prognostic genes were selected for ProgGenes and ProgNGenes sets (bottom-up approach). Differentially expressed genes(DEGs) were selected by comparing the profiles of tumors and non-tumors synchronously (top-down approach). Next, the three gene clusters identified by both approaches went through both network and pathway analysis to obtain most important genes for prognostic signature assembling and model construction. Finally the signatures were validated in one independent clinical data set.

In this study, we have mainly implicated the ‘bottom-up’ approach to develop HCC prognostic signatures both in tumor and adjacent non-tumor tissues. The workflow of our analysis is shown in Fig. 1. In the training cohort, we first identified genes significantly associated with clinical staging systems or patient survival times, then selected some representative and important markers by network and pathway enrichment analyses, finally developed two signatures that can predict overall survival of HCC patients. Likewise, the ‘top-down’ approach have also been tried. Differentially expressed genes(DEGs) in tumor tissues compared to adjacent non-tumor tissues were firstly obtained, then we adopted the same bioinformatics methods to identify some core genes, based on which a DEGs-related signature was finally constructed. The three signatures were further tested in an independent cohort. The results showed that signatures developed by the ‘bottom-up’ approach were more efficient compared to signatures identified by the ‘top-down’ approach, and two signatures identified by ‘bottom-up’ approach were validated in the independent cohort. In addition, we have evaluated the potential application of our molecular markers as drug targets by drug repositioning analysis.

## Materials & Methods

### Datasets: Genomic profiles and patient information

Publicly available datasets with whole-genome gene expression measures in 225 tumors and 220 adjacent non-tumor tissues from 225 primary HCC patients were downloaded from microarray databases Gene Expression Omnibus (GEO: http://www.ncbi.nlm.nih.gov/projects/geo/), the accession number is GSE14520[14]. Pre-processed series of matrixes originally provided by the authors were used in our analysis. Accessory available clinical and follow-up data were also provided by the authors. Patient and tumor features are detailed in Table 1. Among the 225 patients, four patients were excluded due to the lack of survival time. The validation set that included 80 tumors and 82 adjacent non-tumor liver tissues from 82 primary HCC patients was also retrieved from Gene Expression Omnibus, the accession number is GSE10143[9]. For each sample, the expression values of all probes for a given gene were reduced to a single value by taking the average expression value.

**Table 1:** Clinical, histological, molecular data of HCC in training cohort.

Variables	Categories	Total(n = 221)	OS		RFS	
			HR	p-value	HR	p-value
Age	< = 56	112(51%)		0.917		0.256
	>56	109(49%)	1.02(0.67–1.56)		1.23(0.86–1.76)	
Gender	female	30(14%)		0.153		0.019*
	male	191(86%)	1.7(0.82–3.52)		2.17(1.13–4.14)	
	NA	3(1%)				
AFP	< = 300ng/ml	118(53%)		0.017*		0.159
	>300ng/ml	100(45%)	1.68(1.1–2.58)		1.25(0.82–1.91)	
ALT	< = 50U/L	130(59%)		0.726		0.23
	>50U/L	91(41%)	1.08(0.7–1.66)		1.25(0.87–1.78)	
	NA	1(0%)				
Size	< = 5cm	140(63%)		0.002*		0.067
	>5cm	80(36%)	1.94(1.26–2.99)		1.41(0.98–2.04)	
Multinodular	No	176(80%)		0.057		0.428
	Yes	45(20%)	1.59(0.99–2.57)		1.19(0.77–1.84)	
cirrhosis	No	18(8%)		0.032*		0.062
	Yes	203(92%)	4.62(1.14–18.8)		2.18(0.96–4.97)	
	NA	2(1%)				
TNM	I	93(42%)		<0.001**		<0.001**
	II	77(35%)	2.08(1.21–3.58)		1.97(1.29–3.01)	
	III	49(22%)	5.05(2.91–8.79)		3.14(1.97–5.01)	
	NA	2(1%)				
BCLC	0-A	168(76%)		<0.001**		<0.001**
	B-C	51(23%)	3.63(2.33–5.65)		2.78(1.88–4.11)	
	NA	2(1%)				
CLIP	0	97(44%)		<0.001**		0.0017**
	1	74(33%)	1.49(0.86–2.56)		2.21(1.42–3.43)	
	2–5	48(22%)	3.75(2.24–6.3)		1.25(0.82–1.91)	

In univariate analysis, the three staging systems, TNM, BCLC and CLIP, were most significantly associated with overall survival and recurrence-free survival. AFP, a-fetoprotein; ALT, alanine transferase; Prognostic staging system: TNM, Tumor Node Metastasis; BCLC, Barcelona Clinic Liver Cancer; CLIP, Cancer Liver Italian Program. OS, overall survival; RFS, recurrence-free survival; HR: hazard ratio.

**P-value <0.01;

*P-value <0.05.

### ‘Top-down’ approach: identification of differentially expressed genes(DEGs)

As the first step of the ‘top-down’ approach, we identified the most varying genes between tumors and non-tumor tissues using Linear Models for Microarray Data (limma)[22] analysis. Eventually, only the DEGs with a false discovery rate(FDR) < 0.05 and fold change ≥2 were selected.

### ‘Bottom-up’ approach: identification of outcome correlated genes in tumor and adjacent non-tumor tissues

In this study, the ‘Bottom-up’ approach was mainly applied to develop HCC prognostic signatures. To obtain genes with potential prognostic value in tumor and adjacent non-tumor tissues, we evaluated all the genes in two aspects (Fig. 1).

### Survival time related genes

Survival-directed prognostic genes contains recurrence free survival(RFS) and overall survival(OS) related genes respectively. The correlation to RFS or OS of each gene is tested by both univariate Cox proportional hazards regression and supervised principal components analysis (SPCA) [23,24]. For univariate-Cox analysis, genes with a p-value < 0.05 was selected as Cox RFS or OS prognostic genes. In our work, the patient outcomes were used as response variables and the first principal component of SPCA were selected to determine most important score for each gene. Finally, top 2000 most important genes were selected as SPCA RFS or OS prognostic genes. At last, overlapping genes identified by both univariate-Cox and SPCA were considered as RFS or OS prognostic genes.

#### Stage related genes

HCC staging systems are commonly used to indicate outcomes. As noted in Table 1, the three clinical staging systems TNM, BCLC, and CLIP are most significantly associated with RFS and OS. We may reason that genes significantly correlated to TNM, BCLC and CLIP also have prognostic values. Therefore, genes associated with the three clinical staging systems were selected. TNM and CLIP related genes were selected by logistic regression analysis and Kruskal-Wallis test. BCLC related genes were selected using logistic regression analysis and t-test. Genes with a p-value <0.05 were regarded as significant genes.

According to the above analysis, five gene sets were identified in tumor tissues as well as adjacent non-tumor tissues, which are related to RFS, OS, TNM, BCLC or CLIP respectively. Finally genes identified in two of five types of prognostic gene sets were defined as prognostic genes(Fig. 1), which was termed as ProgGenes in tumors. Similarly, a set of prognostic genes termed ProgNGenes in adjacent non-tumors were also determined.

### Ranking of candidate gene sets by network prioritization

#### Networks construction

To identify the key genes involved in HCC from the aforementioned three larger sets of candidate genes separately, we constructed three protein-protein interaction (PPI) networks and performed topological analysis for each. Each gene set was firstly converted to be the seed proteins. Initial interactions among these proteins with a confidence score > 0.4 were obtained from STRING database (version 9.1) (Search Tool for the Retrieval of Interacting Genes/Proteins; http://string.embl.de/). Then, to ensure these interactions truly exist in the gene expression profiles, only gene pairs with a p-value from Pearson Correlation Test less than 0.05 were retained. After that, an undirected network was finally constructed based on these gene pairs.

#### Topological analysis of protein interaction networks

In order to analyze these three networks and to search topologically important nodes, three fundamental measurements in network: degree, betweenness centrality(BC) and closeness centrality(CC) were calculated. Degree measures how many neighbors a node directly links to. A node with high degree centrality may have more influence over others. BC measures how often nodes occur on the shortest paths between other nodes[25], and CC measures the average length from a node to all other nodes[26]. In the PPI network, the nodes with high degree are defined as hub nodes, the nodes with high BC were defined as bottleneck nodes[27], and the nodes with high CC are also important[28], all the three types of nodes are key nodes in the network. In this study, the prioritization of candidate genes was based on the average ranking derived from the three parameters. For each gene in the network, a comprehensive rank score(RS) was calculated as follow:

$$\text{RS}=\text{average}({\text{Rank}}_{\text{degree}}+{\text{Rank}}_{\text{BC}}+{\text{Rank}}_{\text{CC}})$$

Where Rank_degree_, Rank_BC_ and Rank_CC_ represent the rank of the gene in the network according to degree, BC and CC.

We selected the top 5% nodes as the key nodes for later analysis. Network visualizations were generated by Cytoscape[29] and network topological parameters were calculated using the Network Analyzer Cytoscape plugin[30].

### Pathway Enrichment Analysis

By using the online software GSEA(gene set enrichment analysis, http://www.broad.mit.edu/gsea/, v3.87), KEGG pathway enrichment analysis was carried out to find the main functional and metabolic pathways involved in HCC. The most significantly enriched genes (FDR<0.001) were mapped to the corresponding KEGG pathways.

### Integration of network prioritization and pathway enrichment

In order to find vital genes not only playing key roles in networks but also possessing important biological functions in pathways, we integrated the network prioritization result and pathway enrichment result. P-value of hypergeometric probability distribution were calculated to examine whether genes in each significantly enriched pathway were also enriched in key genes selected from PPI network. Significant genes involved in pathways of the same main category were combined into a gene cluster. At last, three types of gene clusters were retrieved, which were representative for DEGs, ProgGenes and ProgNGenes.

### Construction of prognostic signatures

Each gene cluster of DEGs, ProgGenes and ProgNGenes type was tested for its prognostic value separately. Both leave one out cross validation (LOOCV) combined with Cox proportional regression and unsupervised hierarchical clustering were used for patient classification. LOOCV involves using each sample in turn as the validation data for prediction, and the remaining samples as the training set to establish Cox proportional hazards model. The classification of the left-out sample was based on predicted value compared with the mean predicted value of the remaining samples. Finally all samples were divided into two classes, then we evaluate whether the two classes show significantly different overall survival[31]. Unsupervised hierarchical clustering is a more popular classifier in many prognostic studies. Here Ward’s method was applied for clustering[32]. Gene clusters that performed well in both LOOCV and hierarchical clustering method are retained to construct integrated prognostic signature for each type of DEGs, ProgGenes and ProgNGenes.

### Validation in the independent cohort

For each type of DEGs, ProgGenes and ProgNGenes, a best integrated prognostic signature was constructed by combining gene clusters in the same category as a final prognostic signature. To evaluate the consistency and robustness of our signatures, we tested their prognostic power in an independent HCC microarray dataset(GSE10143) using LOOCV and hierarchical clustering. Further, we compared the performance of our signatures with other 21 signatures reported with prognostic value for either recurrence or survival of HCC from Molecular Signature Database (v4.0, http://www.broadinstitute.org/gsea/msigdb).

### Survival Analysis

We used log-rank test and Kaplan-Meier method to assess survival. Survival analyses were performed using the Cox proportional regression model. All statistical analyses were conducted using the open-source R software (http://www.r-project.org). All P values were two sided.

### Drug repositioning analysis

Genes identified by our methods play very important roles in HCC and approved drugs targeting these genes might also be effective for treating HCC. Information on drugs, drug targets and drug indications was obtained through a combination of Therapeutic Target Database(TTD, http://xin.cz3.nus.edu.sg/group/ttd/ttd.asp) and DrugBank (http://www.drugbank.ca/).

## Result

### Identification of top-down and bottom-up candidate prognostic gene sets in HCC

To identify a robust molecular signature to predict outcome of HCC, we analyzed the genome-wide expression profiling of 225 HCC patients from three aspects (see flowchart in Fig. 1).

According to the top-down approach, we first compared gene expression profiles between tumor tissues and adjacent non-tumor tissues. A total of 1400 transcripts corresponding to 1079 genes were selected as DEGs.

In bottom-up approach, 1312 genes were significantly related to outcome or prognostic staging systems within tumor tissues, which were termed as ProgGenes. Similarly, 872 prognostic genes obtained from adjacent non-tumor tissues were regarded as ProgNGenes. In the following analysis, each of the three candidate gene sets will be analyzed to identify a prognostic gene signature separately.

### Prioritization of candidate gene clusters by network topology and function analysis

In order to excavate stable and important genes involved in the development of hepatocellular carcinoma, we combined network and pathway enrichment analysis.

The three candidate gene sets were converted to seed proteins. Then functional linkages among these proteins were acquired from the STRING database. However, these associations were derived through wide diversity of experiments or prediction algorithms, and detected in various types of cell rather than in hepatic cells. Therefore, we next evaluated all the interactive pairs with our expression profiles. Only significantly related pairs were retained to construct subsequent molecular networks. At last, three PPI networks were constructed based on these linkages respectively, as shown in S1 Fig. A-C. The interactions of DEGs network were most closely linked(density: 0.019, and average neighbors: 15.68). And the next is the ProgNGenes network, of which density and average neighbors is 0.009 and 4.96 separately. The density and average neighbors of ProgGenes network is 0.007, 6.35 respectively. To extract key genes from one big network effectively, we analyzed and processed the networks with measurements of degree, betweenness centrality(BC) and closeness centrality(CC). The average ranking of these three measurements for each node in the undirected networks was evaluated. Nodes at top 5% of the networks, which are more important than other less connected nodes, were selected as the core network gene sets (S1 Table).

KEGG pathway enrichment analyses were carried out for the three candidate gene sets separately. The DEGs were significantly enriched in 78 pathways, out of which 28 belong to metabolism related subcategories(S2 Table). Besides, the ProgGenes were significantly enriched in 86 pathways, and 39 out of which also belong to metabolism related subcategories(S3 Table). Relatively, ProgNGenes were enriched in 52 pathways, out of which only 4 related to metabolism, the other enriched pathways belong to 19 subcategories such as signal transduction and immune system(S4 Table).

In order to find vital genes not only playing key roles in networks but also possessing important biological functions, we examined whether genes in each significantly enriched pathway were at the same time enriched in core genes derived from PPI network analysis. The significant genes are shown in S5–S7 Tables. It can be seen that many genes involved in different pathways belong to the same main category. To generate representative signatures for each type of DEGs, ProgGenes, and ProgNGenes, significant genes belonging to the same main category were combined into one functional gene cluster. Eventually, five gene clusters termed as D1-D5 which were most representative for DEGs, four gene clusters P1-P4 for ProgGenes and five clusters PN1-PN5 for ProgNGenes were obtained. In addition, because that immune microenviroment has been proven with value for predicting outcomes[33] and many immune related pathways were identified in non-tumor tissues, we supposed that genes related to immune system to be important for HCC prognosis prediction. These genes were singled out as PN6. Moreover, as we all know, most cancers exhibit similar characteristics and share some common related genes such as EGFR, P53. We hypothesized that our final identified genes embedded in cancer related pathways may also be indicators for HCC outcome, and these genes in DEGs, ProgGenes and ProgNgenes were group as signature D6, P5 and PN7.

### Evaluation of prognosis performance for functional gene clusters and construction of final combined prognostic gene signatures

For our ultimate goal of prognosis analysis for HCC patients, we first assessed the prognosis performance of all the functional gene clusters. We adopted supervised LOOCV and unsupervised hierarchical clustering methods to test whether each gene cluster could classify patients into two groups with significantly different overall survival(OS) rates (good and poor prognosis respectively). The corresponding p-values for each functional gene cluster are shown in Table 2 and S2 Fig. From the table we can see most of the gene clusters show significant prognostic relevance, clusters in non-tumor perform even better than clusters of DEGs and ProgGenes (from tumor). Among all the clusters identified in DEGs and ProgGenes, metabolism related cluster D1, replication and repair cluster D2, cell growth and death cluster D3, carbohydrate metabolism cluster P1, mTOR signaling cluster P2, cancer related cluster P5 were significant (p<0.05) or showed a tendency (p<0.1) for prognostic value for HCC, evaluated by both LOOCV and hierarchical clustering. In non-tumor tissues, one carbon pool by folate cluster PN1, signal transduction cluster PN2, Human Diseases cluster PN5, immune related cluster PN6 and cancer related cluster PN7 were tested to have prognosis value for HCC. These clusters with a small number of genes showed great prognostic potential for hepatocellular carcinoma.

**Table 2:** Log rank p-values computed for all the gene clusters in training set.

Signatures	Size	LOOCV	Hclust
D1(metabolism)	9	0.00356*	0.0308*
D2(Replication and repair)	4	0.00444*	0.0357*
D3(Cell growth and death)	5	0.0899#	0.0937#
D4(Organismal Systems)	4	0.0232*	0.207
D5(Human Diseases)	6	0.119	0.662
D6(cancer related)	5	0.2602	0.5160
P1(Carbohydrate metabolism)	5	0.0181*	0.054#
P2(Environmental Information Processing)	2	0.0468*	0.0854#
P3(Cellular Processes)	4	0.00343*	0.938
P4(human deseases)	14	0.0221*	0.225
P5(cancer related)	7	0.0799#	0.0623#
PN1(One carbon pool by folate)	1	0.00653*	0.00483*
PN2(Signal transduction)	4	0.00424*	0.00593*
PN3(Cell growth and death)	5	0.0433*	0.178
PN4(Organismal Systems)	9	0.0237*	0.108
PN5(Human Diseases)	8	0.00325*	0.00286*
PN6(immune systems or disease)	8	0.00669*	0.0379*
PN7(cancer related)	6	0.0201*	0.0459*

*p < 0.05, represents the signature is significantly related to outcomes

#0.05<p<0.1, represents this signature has a tendency to be related to outcomes

Size: gene number in each gene cluster.

**Fig 2:**
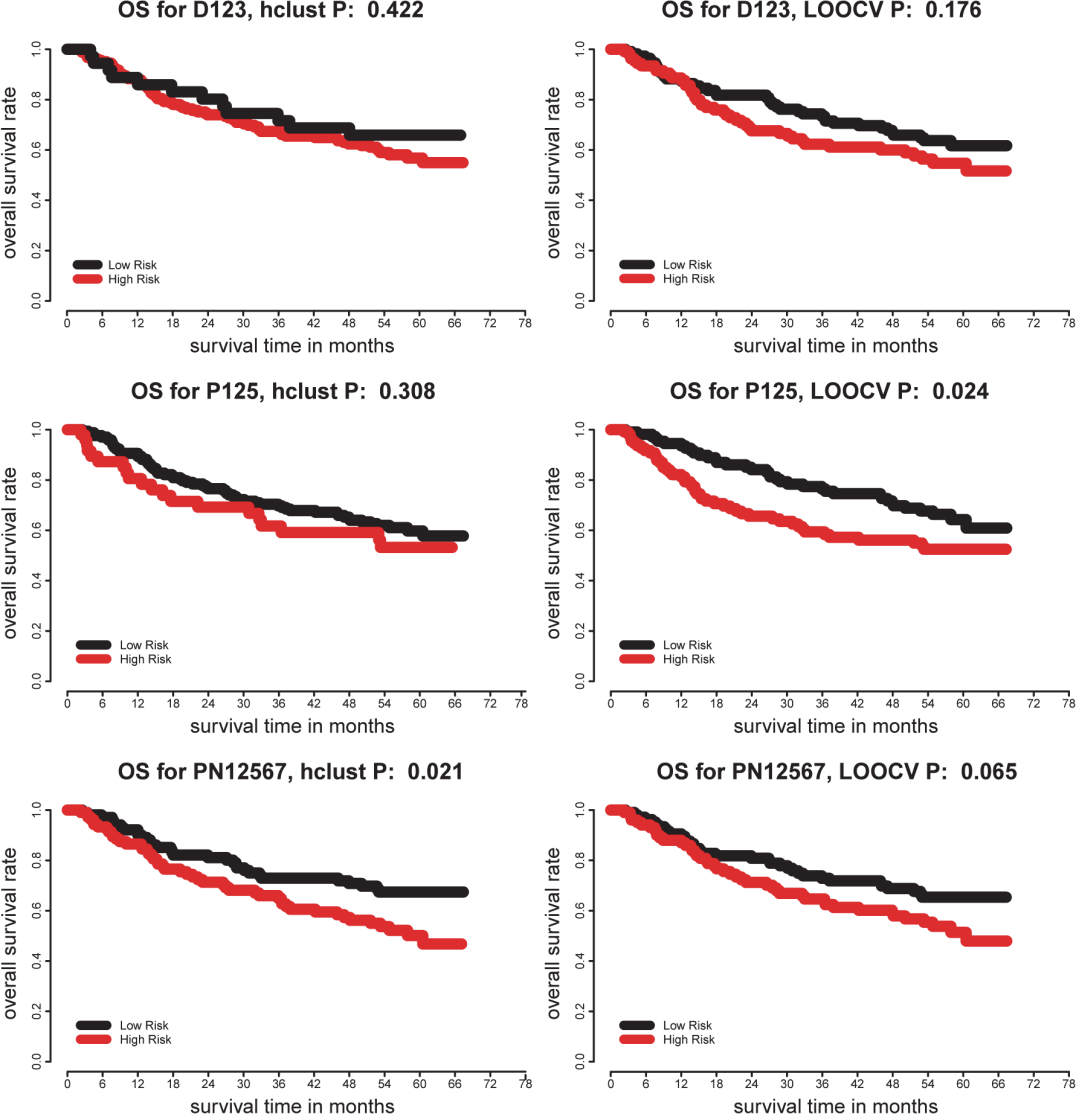
Kaplan-Meier plots of overall survival of individuals with different subtypes from LOOCV and hierarchical clustering analysis for signature D123, P125, PN12567 in training set. A-B, D123 failed to show significant predictive performance according to both LOOCV and HC. C-D, P125 successfully divided patients into two significant groups by LOOCV (p = 0.024) but not by hierarchical clustering. E-F, PN12567 performed well in HC (p = 0.021) and patients classified by LOOCV also showed different OS rates but not significant (p = 0.065).

In order to retrieve a comprehensive and representative prognostic gene signature for each type of DEGs, ProgGenes and ProgNGenes, we combined gene clusters in each type as listed above which possess prognosis values in HCC. At last, three representative prognostic gene signatures were finally constructed, D123 was combined by D1, D2 and D3, P125 was united by P1, P2, P5, and similarly PN1, PN2, PN5, PN6, PN7 were combined into PN12567. Next we reexamined the prognosis power of these representative three signatures in the original patient cohort of HCC. The clustering result for D123, P125 and PN12567 was shown in S3 Fig. It can be seen from the survival curves shown in Fig. 2A-B, D123 failed to show significant predictive performance according to both LOOCV and HC. P125 successfully divided patients into two significant groups by LOOCV (p = 0.024) but not by hierarchical clustering(Fig. 2C-D). PN12567 performed well in HC (p = 0.021, Fig. 2E) and patients classified by LOOCV also showed different OS rates but not significant (p = 0.065, Fig. 2F).

### Validation of prognostic gene signatures from DEGs, ProgGenes and ProgNGenes in one independent cohort

**Fig 3:**
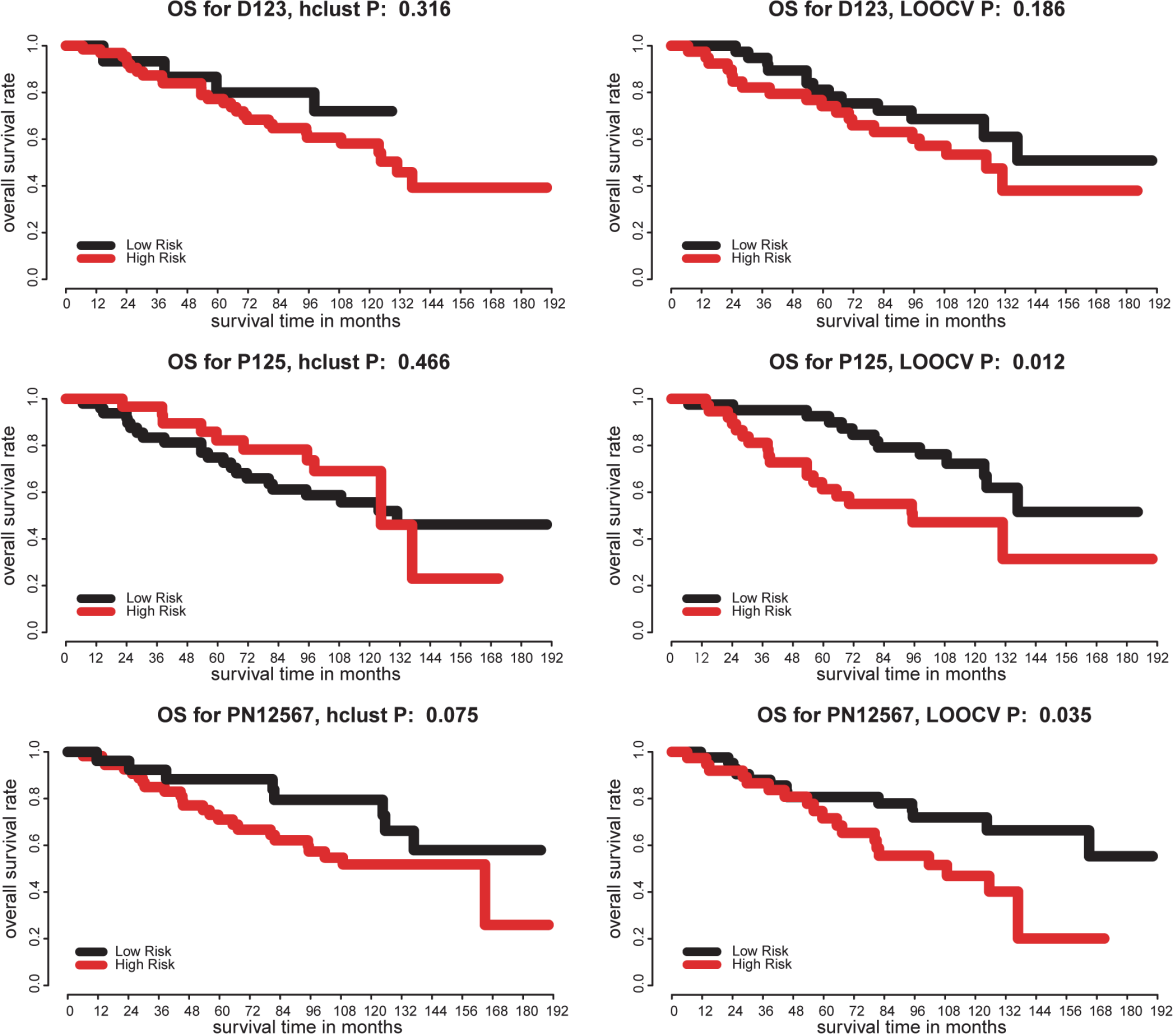
Prognosis analysis based on signature D123, P125, PN12567 in independent validation set. A-B, predictive performance of D123 according to both LOOCV and HC. C-D, P125 successfully divided patients into two significant groups by LOOCV (p = 0.012) but not by hierarchical clustering. E-F, PN12567 performed well in LOOCV (p = 0.035) and patients classified by HC also showed different OS rates but not significant (p = 0.075).

We further evaluated the prognostic power of the three final integrated gene signatures from DEGs, ProgGenes and ProgNGenes respectively in an independent set of 82 HCC tumor and adjacent non-tumor tissue samples. S4 Fig. showed the clustering result for D123, P125 and PN12567. The results of LOOCV and hierarchical clustering are shown in Fig. 3A-F. The top-down signature D123 failed to show significant predictive performance. The two bottom-up signatures P125 and PN12567 could significantly predict overall survival by LOOCV(P = 0.012, 0.035, Fig. 3C, E), but not by hierarchical clustering (P = 0.466, 0.075, Fig. 3D, F).

Furthermore, we compared our results with the performances of 21 reported signatures related to tumor recurrence, metastasis or survival in HCC. Most of these reported signatures were derived from DEGs and analyzed by the pipeline of top-down approaches. None of them successfully classified patients into two groups with significantly different overall survival by LOOCV(S8 Table). These results suggest that prognostic gene signature identified by bottom up approaches might be more robust than those identified by top-down approaches, and also contain fewer genes.

### Indications of prognostic markers in drug-repositioning

After all the above analyses, we have finally obtained two prognostic gene signatures P125 and PN12567 for HCC patients. For decades, only a few drugs have been effective for the treatment of hepatocellular cancer such as the widely used Sorafenib. We investigated the potential of identifying indications to HCC for existing drugs based on these prognostic biomarkers, since they have been proven to be consistently correlated with HCC patients outcome. By scanning the Therapeutic Target Database and DrugBank[34,35], we found that 10 out of 11 genes in P125 and 10 out of 14 genes in PN12567 are annotated as druggable targets (S9 Table). These proportions are much higher than if derived from the whole genome, which contains 19027 protein coding genes and 3421 available druggable targets(18%, p < 7e^-9^ for P125 and p < 2e^-6^ for PN12567). By retrieving the DrugBank database, we furthermore found that 11 human drug targets(8 for P125, 3 for PN12567) can be modulated by 40 unique approved drugs(Table 3). However, all the indications of these approved drugs were unrelated to hepatocellular cancer before, which means, they might be repositioned to HCC-related as well. Meanwhile, there are 3 genes in P125 and 11 genes in PN12567 left which are not annotated by DrugBank. Furthermore, we studied their interactions with drug targets by analysis of the subnetworks of P125 and PN12567. Interestingly, all the genes in each signature set were connected closely (S5 Fig.). These results suggested that some signatures which play important roles in HCC tumorigenesis and are not directly targeted by drugs, such as HIF1A, IGF1 in P125 subnetwork and TP53, PIK3CB in PN12567 subnetwork, were the direct/indirect interactive genes of drug targets.

**Table 3:** Eleven Successfully searched human drug targets(8 for P125, 3 for PN12567) are modulated by 40 unique approved drugs.

Genes	DrugBank ID	Name	TTD ID	Disease
PKLR	DB00119	Pyruvic acid	DAP000543	Dietary shortage
PKM2	DB00119	Pyruvic acid	DAP000543	Dietary shortage
MDH2	DB00157	NADH	DAP001291	Parkinson's disease
SDHB	DB00139	Succinic acid	DAP000545	Dietary shortage
PPARG	DB00159	Icosapent	DAP000969	Hyperglyceridemic
	DB00244	Mesalazine	DAP000729	Ulcerative colitis
	DB00328	Indomethacin	DAP000617	Patent ductus arteriosus
	DB00412	Rosiglitazone	DAP000271	Diabetes mellitus
	DB00731	Nateglinide	DAP000918	Diabetes mellitus
	DB00795	Sulfasalazine	DAP000153	Inflammatory bowel dieaseas and rheumatoid arthritis
	DB00912	Repaglinide	DAP000133	diabetes mellitus
	DB00966	Telmisartan	DAP000766	diabetes mellitus
	DB01014	Balsalazide	DAP000733	Inflammatory bowel disease
	DB01050	Ibuprofen	DAP000780	Pain
	DB01067	Glipizide	DAP000920	diabetes mellitus
	DB01132	Pioglitazone	DAP000272	diabetes mellitus
	DB01252	Mitiglinide	DAP000917	diabetes mellitus
	DB01393	Bezafibrate	DAP001182	hyperlipidaemia
EGFR	DB00072	Trastuzumab	DAP000391	breast cancer
	DB00281	Lidocaine	DAP000121	anesthetic
	DB00317	Gefitinib	DAP000657	cancers
	DB00530	Erlotinib	DAP001010	non-small cell lung cancer
	DB01259	Tykerb	DCL000344	bladder, head & neck, nsclc, brain cancer
JUN	DB00570	Vinblastine	DAP000785	cancers
	DB01029	Irbesartan	DAP000364	hypertension
HSP90AA1	DB00615	Rifabutin	DAP000656	tuberculosis and mycobacterium avium complex (mac) disease
TYMS	DB00293	Raltitrexed	DAP000759	colon and rectum cancers
	DB00322	Floxuridine/5-fluorouracil	DAP001245	colorectal cancer
	DB00432	Trifluridine	DAP000760	viral infection
	DB00440	Trimethoprim	DAP000927	urinary tract infections
	DB00441	Gemcitabine	DAP001246	cancers
	DB00544	Fluorouracil	DAP000829	cancers
	DB00642	LY231514	DCL000320	non-squamous non-small cell lung cancer
	DB00650	Leucovorin/5-fluorouracil	DAP001244	colon cancer
	DB01101	Capecitabine	DAP000761	colorectal cancer
HDAC2	DB00227	Lovastatin	DAP000551	hypercholesterolemia
	DB00277	Theophylline	DAP000002	chronic obstructive pulmonary disease
	DB01223	Aminophylline	DAP000613	bronchial asthma
	DB01303	Oxtriphylline	DAP000868	cough
	DB02546	Vorinostat	DAP001082	cutaneous t-cell lymphoma
VCAM1	DB01136	Carvedilol	DAP000135	congestive heart failure

## Discussion

Prognosis analysis is probably the most frequently performed clinical modeling in cancer research based on gene expression profiles. A top-down approach pipeline is quite conceivable, that is, first differentially expressed genes(DEGs) in cancer versus control were identified by statistical methods, then certain gene prioritization methods were utilized to reduce the DEGs to a smaller gene signature set that possess the best prognostic power, finally appropriate classification signatures were constructed for prognosis analysis or prediction based on this gene signature of clinically applicable size. Multiple choices of methods exist for each step in the pipeline, which should be carefully evaluated and decided. For examples, gene prioritization can be done through regression correlation, network topology, pathway enrichment, GO enrichment, key network modules, combinatorial regulatory network s et al[36,37].

In this work, we emphasize another direction, the bottom-up approach for prognostic signature identification based on gene expression profiles. It is more like an objective-oriented approach. That is to say, in a training set with patient samples and survival information and gene expression datasets, outcome(survival time, tumor stage) correlated genes were statistically identified in the first step, then prognostic gene signatures formed by core functional genes were extracted by gene prioritization, finally clinical prognostic signatures were constructed based on prioritized gene signatures. The established prognostic signatures must be validated in independent datasets. In this work gene prioritization utilized combination of network topology and pathway enrichment.

The finding that adjacent non-tumor tissues to hepatocellular cancer actually showed strong tendency for prognosis may have several biological implications. First it states for the importance of tumor surrounding environment. How well a tumor patient will survive may actually be determined on how adverse the tumor surrounding environment is, rather than or in addition to how adverse the tumor itself is. Second it provides the molecular base for strong pathological value of adjacent tissue to HCC which has been clinically repeatedly confirmed with long-term inflammation and cirrhosis.

Because the prognostic gene signatures identified from bottom-up approach are certainly related to patient outcome such as survival time and tumor stage, they must be genes that are important in tumor development and progression. For examples, IGF1 reflects hepatic synthetic function and takes part in the development and progression of various cancers, and low IGF1 level indicates a poor prognosis in hepatocellular carcinoma[38,39]; HIF1A plays an essential role in embryonic vascularization, tumor angiogenesis and its overexpression is related to an unfavorable prognosis in HCC[40,41]; PPARG involved in PPAR signaling pathway exerts an inhibitory effect on tumor cell growth in hepatocellular carcinoma and therefore its dysregulation affects HCC outcome[42,43]; EGFR plays prominent roles in HCC proliferation, and metastasis[44]. CXCR4 is a comprehensive cytokine receptor with an important role in the dissemination and metastasis in HCC[45,46]; Another immune response gene H2AFX is reported to be associated with fibrosis progression and hepatocarcinogenesis[47]. It should be rational to consider them as good candidate targets for intervention. Previously, we have integrated mutation data from various sources to find significantly mutated genes in HCC[48]. Here in order to find direct genomic variation evidence for our signature genes, we downloaded 202 HCC patients’ mutation information from TCGA (http://cancergenome.nih.gov/) to find high frequently mutated genes which might play important roles in HCC pathogenesis. Only the mutated frequencies of EGFR and HSP90AA1 in P125 reached 5%. And they have been targeted by approved drugs (see Table 3). Therefore it might be rational to search for other druggable targets.

We therefore in our work made a preliminary try to investigate the potential of using such prognostic genes to guide drug-repositioning. To our expectation, a very high percentage of the prognostic gene signatures are druggable targets which have been indicated by approved drugs but not having been referred to hepatocellular cancer. For examples, PPAGR, included in P125, is a target for a variety of drugs. Current indication for Rosiglitazone (DrugBank ID: DB00412) that targets PPAGR is diabetes mellitus. Rosiglitazone appears to activate PPAGR and has an anti-inflammatory effect that down-regulates nuclear factor kappa-B (NFκB) level[49,50]; Sulfasalazine(SASP, DB00795), another PPAGR-targeted drug, for which the current indication is inflammatory bowel disease and rheumatoid arthritis, is also useful in restricting lymphoma growth and for therapy of prostate cancers by inhibiting cystine transporter[51,52]; Telmisartan (DB00966) is effective for prevention and treatment of prostate cancer[53]. These observations imply that Rosiglitazone, Sulfasalazine and Telmisartan could have alternative indications for intervention of hepatocellular carcinoma. Another representative marker TYMS in PN12567, plays an important role in DNA methylation, synthesis, and repair. Fluorouracil(DB00544) targeting TYMS is an antineoplastic anti-metabolite, which could masquerade as purine or pyrimidine and form the building blocks of DNA and result in DNA synthesis being inhibited. Fluorouracil injection is indicated in the palliative management of some types of cancer, including colon, breast, renal cell and so on[54]. It was reported that enhancement of TYMS is also related to a reduced risk of HCC through discouraging the misincorporation of uracil into DNA, and some HCC patients may be benefited from 5-Fluorouracil-based chemotherapy[55,56]. Similarly, Gemcitabine(DB00441) inhibits thymidylate synthetase, leading to inhibition of DNA synthesis and cell death, is indicated for the treatment of advanced cancers[57]. These reports also support that Fluorouracil and Gemcitabine may be implicated in drug-repositioning for HCC.

The main limitation to this study is that the training cohort was not big enough or representative enough. Therefore the carefully elaborated prognostic gene signatures although can be validated in one or more independent patient cohort(s), it is hard to expect them to possess universal values for prognosis for HCC patients in general. The road to identifying as good prognostic features in hepatocellular cancers as in breast cancers is still long. Identifying common prognostic signatures is more difficult for HCC due to the high heterogeneity as well as the population diversity. Our work represented by bottom-up prognostic gene signature identification and drug-repositioning analysis may be proved as useful approaches for HCC outcome study and intervention, after being tested on more datasets in the future.

## Supporting Information

S1 Fig Networks base on the three candidate gene sets. The dissociated nodes have been removed. (A) Network built by DEGs containing 843 nodes and 6610 edges. (B) Network of ProgGenes containing 892 nodes 2830 edges and (C) Network of ProgNgenes was consisted of 539 nodes and 1366 edges. Nodes shades of red to green color represented high to low BC values.
(TIF)

S2 Fig Prognosis performance of the 17 gene clusters in training set. D1, D2, D3, P1, P2, P5 and PN1, PN2, PN5, PN6, PN7 were significant (p<0.05) or showed a tendency (p<0.1) for prognostic value for HCC, evaluated by both LOOCV and hierarchical clustering.
(TIF)

S3 Fig The hierarchical clustering result for D123, P125 and PN12567 in training cohort. (TIF)

S4 Fig The hierarchical clustering result for D123, P125 and PN12567 in validation cohort. (TIF)

S5 Fig The subnetworks which only including genes in P125(A) and PN12567(B). The green nodes represent the known drug targets and yellow nodes represent the gene signatures which were not annotated in the DrugBank and TTD databases, respectively.
(TIF)

S1 Table The three core gene sets representative for DEGs, ProgGenes and ProgNGenes by network analysis. Genes at top 5% of the networks, which should be more important than other less connected nodes, were selected as the core network gene sets.
(XLSX)

S2 Table KEGG pathway enrichment analysis for DEGs. (XLSX)

S3 Table KEGG pathway enrichment analysis for ProgGenes. (XLSX)

S4 Table KEGG pathway enrichment analysis for ProgNGenes. (XLSX)

S5 Table Six gene clusters D1-D6 identified from DEGs. (XLSX)

S6 Table Five gene clusters P1-P5 identified from ProgGenes. (XLSX)

S7 Table Seven gene clusters PN1-PN7 identified from ProgNGenes. (XLSX)

S8 Table Log rank p-values for the 21 reported signatures. None of them successfully classified patients into two groups with significantly different overall survival by LOOCV in the validation set.
(XLSX)

S9 Table Genes annotated as druggable targets in P125 and PN12567. 10 out of 11 genes in P125 and 10 out of 14 genes in PN12567 are potential drug targets.
(XLSX)
