# Supplementary figures and images for: Identifying Prognostic Features by Bottom-Up Approach and Correlating to Drug Repositioning
Source: PLoS One. 2015 Mar 4;10(3):e0118672. doi: 10.1371/journal.pone.0118672 (PMC4349868; doi:10.1371/journal.pone.0118672)

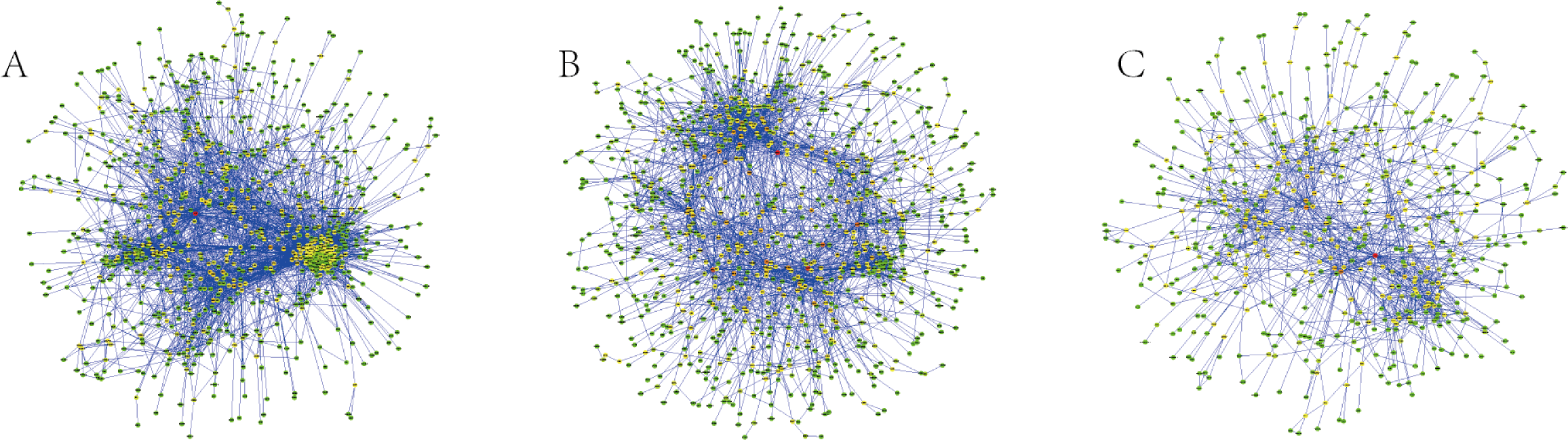

Supplement: S1 Fig — The dissociated nodes have been removed. (A) Network built by DEGs containing 843 nodes and 6610 edges. (B) Network of ProgGenes containing 892 nodes 2830 edges and (C) Network of ProgNgenes was consisted of 539 nodes and 1366 edges. Nodes shades of red to green color represented high to low BC values. (TIF) [file pone.0118672.s001.tif]

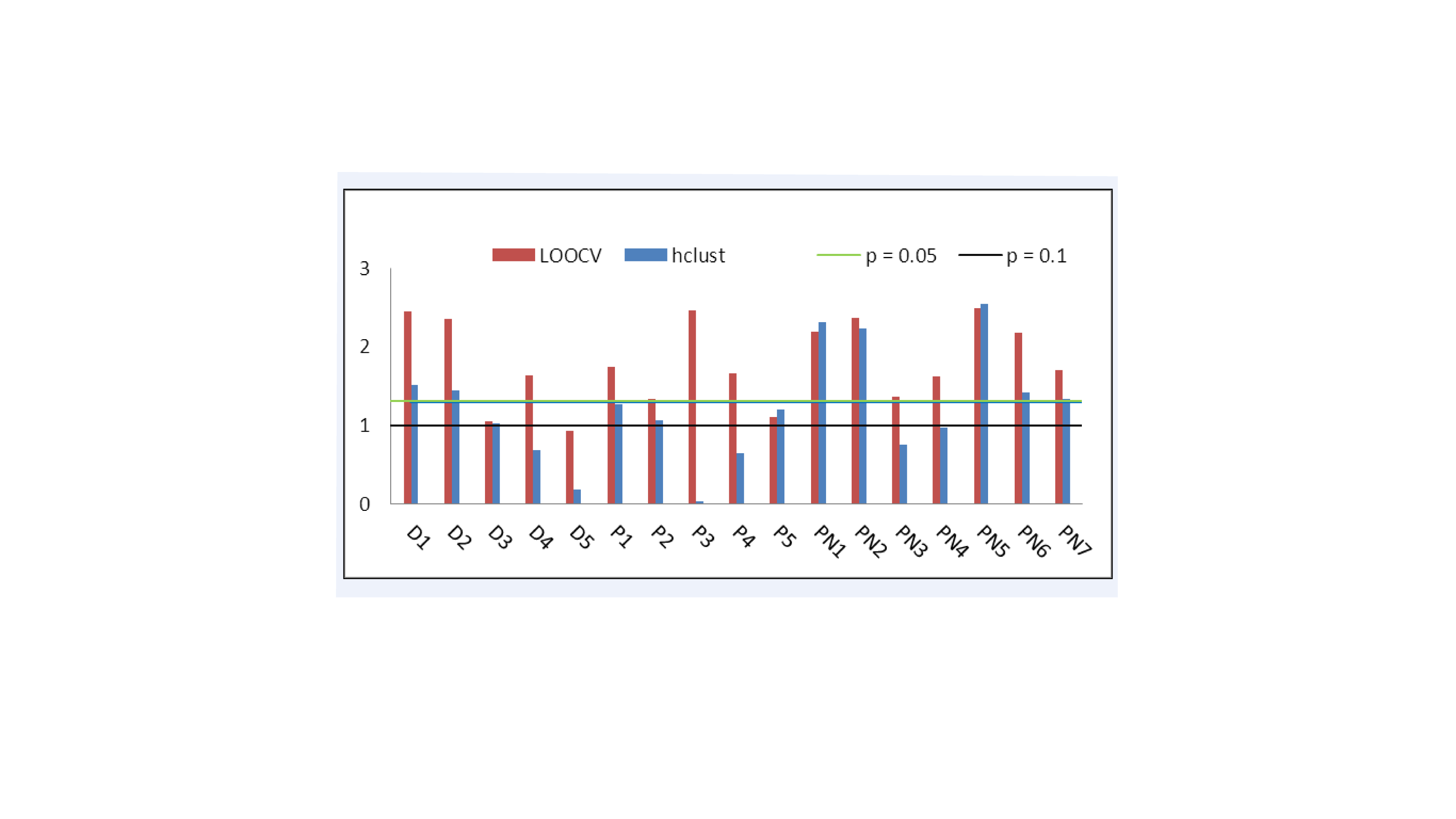

Supplement: S2 Fig — D1, D2, D3, P1, P2, P5 and PN1, PN2, PN5, PN6, PN7 were significant (p<0.05) or showed a tendency (p<0.1) for prognostic value for HCC, evaluated by both LOOCV and hierarchical clustering. (TIF) [file pone.0118672.s002.tif]

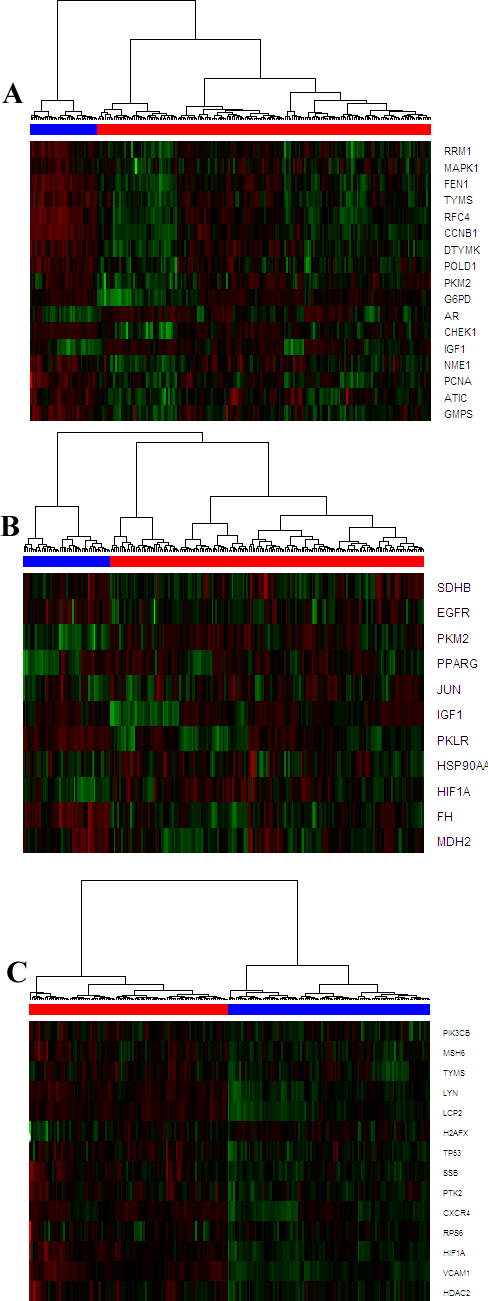

Supplement: S3 Fig — (TIF) [file pone.0118672.s003.tif]

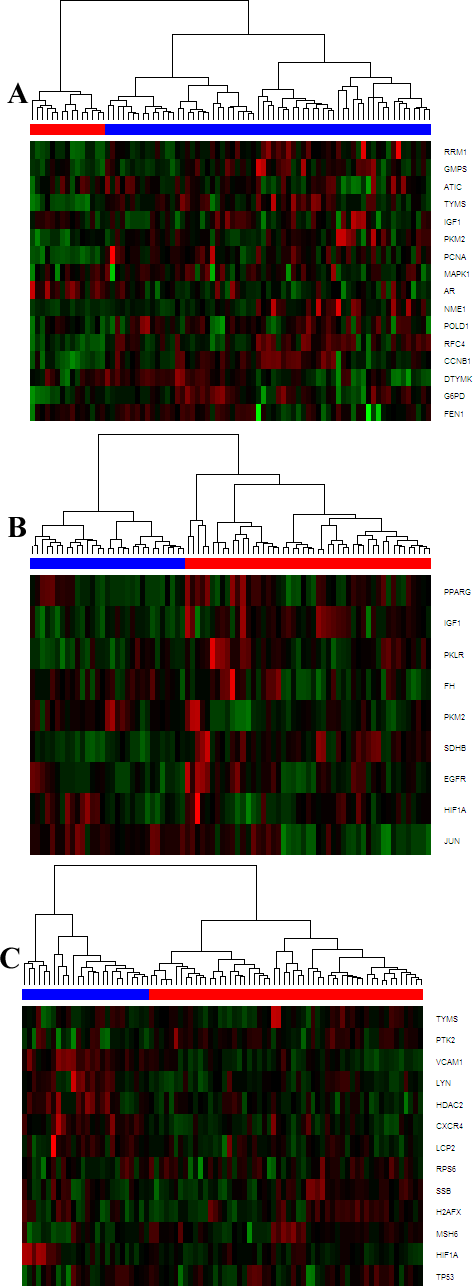

Supplement: S4 Fig — (TIF) [file pone.0118672.s004.tif]

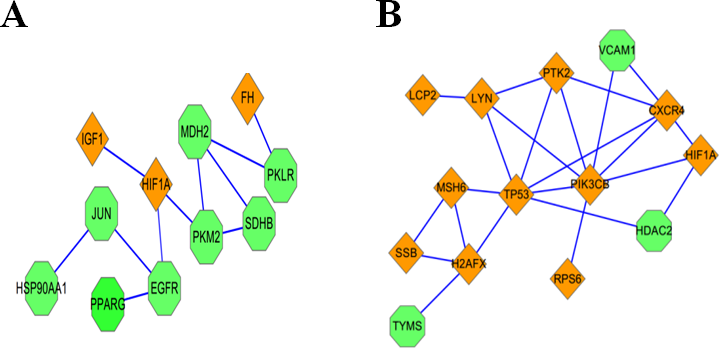

Supplement: S5 Fig — The green nodes represent the known drug targets and yellow nodes represent the gene signatures which were not annotated in the DrugBank and TTD databases, respectively. (TIF) [file pone.0118672.s005.tif]
